# A lightweight sensing platform for monitoring sleep quality and posture: a simulated validation study

**DOI:** 10.1186/s40001-018-0326-9

**Published:** 2018-05-30

**Authors:** Richard M. Kwasnicki, George W. V. Cross, Luke Geoghegan, Zhiqiang Zhang, Peter Reilly, Ara Darzi, Guang Zhong Yang, Roger Emery

**Affiliations:** 1 Department of Surgery and Cancer, Imperial College London, 10th Floor QEQM Building, St. Mary’s Hospital, Praed Street, London, W2 1NY UK; 2 The Hamlyn Centre, Institute of Global Health Innovation, Imperial College London, London, UK; 3 Imperial College Healthcare NHS Trust, London, UK

**Keywords:** Shoulder, Pervasive, Monitoring, Posture, Sleep, Activity, Sensors, Wearables

## Abstract

**Background:**

The prevalence of self-reported shoulder pain in the UK has been estimated at 16%. This has been linked with significant sleep disturbance. It is possible that this relationship is bidirectional, with both symptoms capable of causing the other. Within the field of sleep monitoring, there is a requirement for a mobile and unobtrusive device capable of monitoring sleep posture and quality. This study investigates the feasibility of a wearable sleep system (WSS) in accurately detecting sleeping posture and physical activity.

**Methods:**

Sixteen healthy subjects were recruited and fitted with three wearable inertial sensors on the trunk and forearms. Ten participants were entered into a ‘Posture’ protocol; assuming a series of common sleeping postures in a simulated bedroom. Five participants completed an ‘Activity’ protocol, in which a triphasic simulated sleep was performed including awake, sleep and REM phases. A combined sleep posture and activity protocol was then conducted as a ‘Proof of Concept’ model. Data were used to train a posture detection algorithm, and added to activity to predict sleep phase. Classification accuracy of the WSS was measured during the simulations.

**Results:**

The WSS was found to have an overall accuracy of 99.5% in detection of four major postures, and 92.5% in the detection of eight minor postures. Prediction of sleep phase using activity measurements was accurate in 97.3% of the simulations. The ability of the system to accurately detect both posture and activity enabled the design of a conceptual layout for a user-friendly tablet application.

**Conclusions:**

The study presents a pervasive wearable sensor platform, which can accurately detect both sleeping posture and activity in non-specialised environments. The extent and accuracy of sleep metrics available advances the current state-of-the-art technology. This has potential diagnostic implications in musculoskeletal pathology and with the addition of alerts may provide therapeutic value in a range of areas including the prevention of pressure sores.

**Electronic supplementary material:**

The online version of this article (10.1186/s40001-018-0326-9) contains supplementary material, which is available to authorized users.

## Background

Pathologies affecting the shoulder are common within the population, often leading to a significant loss of function. UK prevalence of self-reported shoulder pain has been estimated at 16%, with rates as high as 26% in the elderly [1]. Accounting for 2.36% of presentations to general practitioners, it is the third most common musculoskeletal presenting complaint [2]. Shoulder pain has been found to lead to significant sleep disturbance, which consequentially has significant impact on quality of life. Further, sleeping posture has been implicated as a causative mechanism of certain shoulder pathologies, as well as having a detrimental impact on post-operative healing following musculoskeletal surgery.

Patients following shoulder surgery demonstrated greater pain intensity and duration in comparison to total hip and knee arthroplasty patient cohorts, with such pain having significantly greater interference with sleep and activities of daily living [3]. Smith et al. [4] demonstrated that 90% of patients were unable to sleep on their affected shoulder post-operatively, additionally 80% of patients with shoulder conditions without prior surgery were also unable to sleep on their affected shoulder [5].

It has been hypothesised that a lateral decubitus sleeping position can lead to increased shoulder pressure for extended periods of time, precipitating chronic pain [6]. Kempf et al. [7] demonstrated a significant correlation of 68% between the side of shoulder pain and preferred sleeping side, and hypothesised that manipulating sleeping posture could prevent further damage to the shoulder. Werner et al. [8] reported that the supine position resulted in significantly lower subacromial pressures compared to prone and lateral decubitus positions, demonstrating that sleep positions leading to active flexion, abduction and internal rotation should be avoided during recovery.

Monitoring sleep quality may provide broad insight into general health status of patients [9–13], with extension of monitoring into patients with shoulder pathologies seeming logical given the high prevalence of sleep disturbance, anxiety and depression [14]. In the setting of shoulder pathology, sleep activity and posture provide surrogate markers for sleep quality and limb positioning. The current gold standard for sleep monitoring is polysomnography (PSG); however, there have been technological advancements to produce less intrusive methods of monitoring sleep, which can be used in a more natural sleep setting. These include actigraphy (physical activity), heart rate variability (HRV), and smartphone applications. Actigraphy and HRV are currently the only methods that have been validated to measure sleep quality to a high degree of accuracy, ranging from 82–97 and 57–93%, respectively [15–21].

Alongside sleep quality, posture monitoring during sleep provides clinically valuable information regarding the arrangement of limbs and resultant joint angles and likely pressures. Electrocardiogram (ECG) waveforms, pressure sensors and ultra-wide band (UWB) frequency technology have all previously been used in the determination of sleep posture, though their efficacy in the patients with shoulder pain is yet to be established [22–27]. Joint angle measurements provide another dimension to sleep posture monitoring and have demonstrated efficacy in the orthopaedic patient cohort. The current main methodologies in the measurement of joint angles include inertial sensors, universal goniometer and smartphone applications, with inertial sensors attracting the greatest interest within the literature [28–34]. However, traditional emphasis has been placed upon the refinement of wearable sensors in isolation. In contrast, the current work aimed to develop a multi-sensor system architecture combining wearable sensors, communication technology and data analytics in a single platform capable of ambulatory monitoring. Herein, the presented study aims to investigate the accuracy of a wearable sleep system (WSS) in the detection of sleep quality and posture.

## Methods

### Study overview

A series of laboratory-based simulations were designed to assess the ability of WSS to detect sleeping posture and activity. Healthy adult subjects were recruited locally at the Hamlyn Centre (Imperial College London) and excluded if they demonstrated active shoulder pathology or reported upper body mobility limitation of any sort. Ethical approval was gained from the NRES Committee London—Dulwich on 19th November 2013 (REC Reference: 10/H0808/124). Informed consent was obtained directly from all study participants.

Sleeping posture was investigated by asking healthy individuals to assume a variety of predefined sleeping positions. Sleep activity was investigated by asking subjects to simulate a typical sleeping period progressing through stages of sleep and replicating associated activity at each stage.

### Wearable sleep system (WSS)

**Fig. 1:**
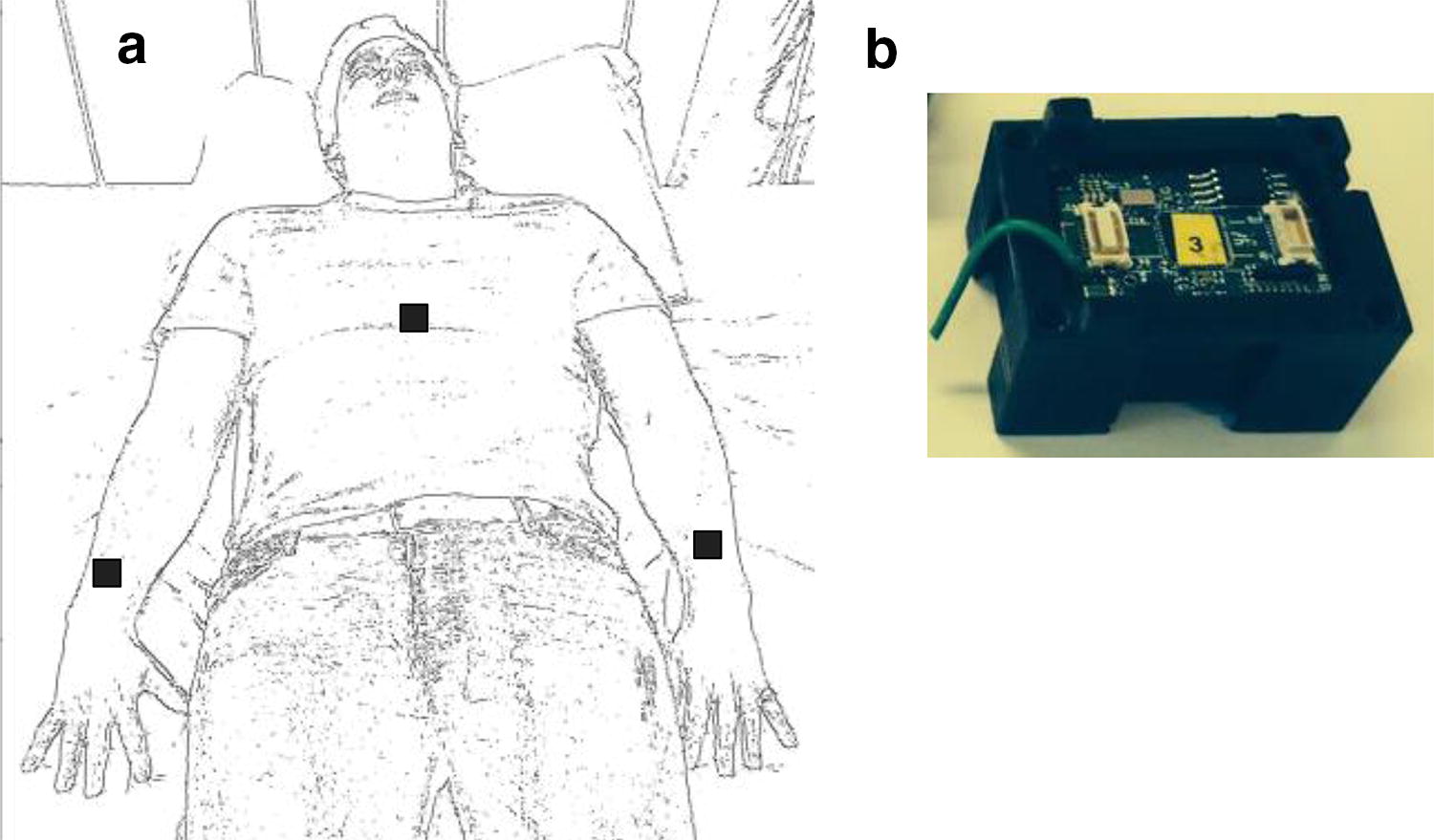
The WSS sensor platform. **a** Schematic representation of WSS sensors placement, with one on each arm and one on the trunk. **b** Structural representation of the WSS sensors used

A bespoke WSS was designed consisting of a small wearable sensor positioned on each arm and the chest, (seen in Fig. 1) communicating wirelessly to a local processing unit (in this study was a laptop computer) using a radiofrequency transceiver. The node platform consists of a TI MSP430 ultra low power processor, a Chipcon CC2420 RF module for wireless communications and a light weight Li-ion polymer battery. The node is integrated with an Analog Devices ADXL330 for measurement of 3D acceleration, an InvenSense ITG-3200 digital gyroscope for 3D angular velocity measurement and a Honeywell HMC5843 for 3D magnetic field measurement as previously described. The whole sensor node measures 20 × 30 × 17 mm with a weight of 10 g and has previously been shown effective in the quantitative analysis of human body movements [35].

### Posture monitoring

**Fig. 2:**
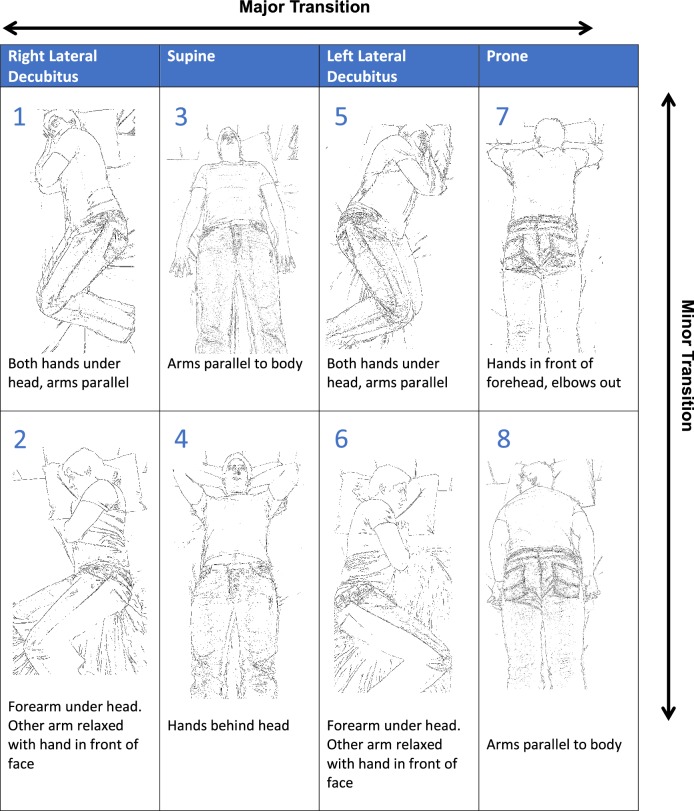
Simulated sleeping postures. (1) Right lateral decubitus—both hands under cheek, arms parallel; (2) right lateral decubitus—bottom forearm under head, top arm relaxed with hand in front of face; (3) supine—arms parallel to body; (4) supine—hands behind head; (5) left lateral decubitus—both hands under cheek, arms parallel; (6) left lateral decubitus—bottom forearm under head, top arm relaxed with hand in front of face; (7) prone—hands in front of forehead; (8) prone—arms parallel to body

Participants were first asked to lie on the bed and assume eight postures in a known order, demonstrated in Fig. 2, to allow sensor calibration. Each posture was maintained for 10 s before participants were instructed to assume the next predefined posture. To facilitate an assessment of blinded classification accuracy, each participant re-enacted the postures in a randomised order. The randomised sequence consisted of 16 total postures with each original posture performed twice by participants. A broad range of postures were chosen to determine the ability of the WSS to recognise both major and minor movements that may be employed by patients in the clinical setting. In order to direct the selection of such positions, focus groups were held with study participants to identify common sleeping positions amongst individuals without shoulder pathology. Further sleeping positions in those with shoulder pain were identified by clinical authors through consultation with patients suffering with shoulder pathologies.

### Sleep quality monitoring

Participants were asked to perform a semi-structured simulation of the three sleep stages (awake, sleep and REM sleep) based upon the degree of major and minor transition movement permitted as outlined in the sleep stage simulation protocol, Table 1. Major transitions were defined as movement between the four main postures: right slide, left side, supine and prone. Minor transitions were defined as isolated movement of the limbs within each of the four main postures. Major and minor transition movements were standardised before recording commenced through verbal explanation and the use of a visual aid, Fig. 2. Each sleep stage was simulated for 2 min with a sequential order of A, B, C, B, C, B, A, providing 14 min of recording activity for each participant, where A represents awake, B represents sleep, and C represents REM sleep. Our string of movements therefore simulates progression throughout natural sleep. Table 1 outlines the degree of movement permitted in each of the sleep stages A, B and C. The sleep stage simulation protocol was developed in view of the limited battery life of the WSS (14 min) and was designed to broadly simulate differing stages of sleep across the constrained time period.

**Table 1:** Sleep stage simulation protocol

Simulated sleep stage	Major transition permitted	Minor transition permitted	Movement between transitions permitted
Awake	Every 30 s	No	Yes
Sleep	No	Every minute	Limited
REM sleep	No	No	No

The degree of movement permitted during the each of the simulated sleep stages is outlined along with corresponding major and minor transition frequencies where appropriate

### Combined proof of concept model

A single participant completed a simulation including combination of both posture and activity changes to assess the validity of WSS as a future platform for total sleep monitoring. Sensors were placed as described in Fig. 1 and the participant was asked to calibrate the sensors with known body position as previously described. Immediately after completion of all eight postures the subject was then asked to perform the same three simulated sleep phases.

### Data analysis

Data from tri-axial accelerometer, gyroscope, and magnetometer for all three sensors were integrated and wirelessly sent to a receiving laptop. An algorithm was developed to allow the training of a computerised posture classifier based on calibration data (see Additional file 1). Data were analysed on MATLAB^®^ (*MathWorks Version R2014a (8.3.0.532)* to quantify both individual and overall accuracy and error. For activity, a surrogate for sleep phase, accelerometer values from each of the three sensors were combined to estimate activity level using data variance, i.e. the magnitude of 3-dimensional movement. Subject-specific activity thresholds were derived from study data and used to determine the simulated sleep phase. The nested IF function: = IF (*F*3 > 0.14, “3”, IF (*F*3 > 0.058, “2”, IF (*F*3 < 0.058, “1”))) was used to quantify activity levels and corresponding sleep stages. To calculate the percentage of time, the participant spent in each phase the COUNTIF function = COUNTIF(H3:H24509,3) was run across all data.

### Statistical analysis

Statistical tests were used to determine if any of the participants or postures demonstrated particularly high and low accuracy levels. The non-parametric test Kruskal–Wallis one-way non-parametric analysis of variance (KW-ANOVA) was used to initially detect any outliers. Outliers were further compared against group averages using the Mann–Whitney *U* Test. Analyses were performed using SPSS version 20.0 for Windows. The statistical significance level was set at *P* < 0.05.

## Results

Sixteen healthy subjects were recruited, ten participating in the posture protocol, five in the activity protocol, and one for the proof on concept simulation. There were seven females and nine males with a mean age of 25 years old. No major technical issues arose and datasets were available for all participants recruited.

### Posture

**Fig. 3:**
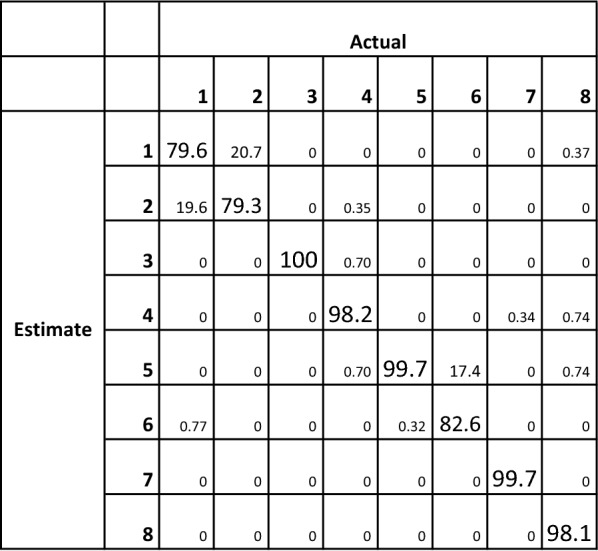
Classification matrix representing the percentage accuracy for the eight main postures

Across 10 participants, the WSS platform showed an overall classification accuracy of 99.5% for detecting the four main sleeping postures: right, supine, left, and prone. Classification accuracy across all eight postures was 92.5% (Fig. 3).

The distinction between postures 1 and 2 was the most difficult to classify, followed by postures 5 and 6. After KW-ANOVA, the mean rank suggested postures 1, 2 and 6 to be outliers. A Mann–Whitney *U* test found no statistical significance between the classification accuracy of these postures compared to the others. Between participants, the classification accuracy varied from 84.3 to 100%.

### Activity

**Fig. 4:**
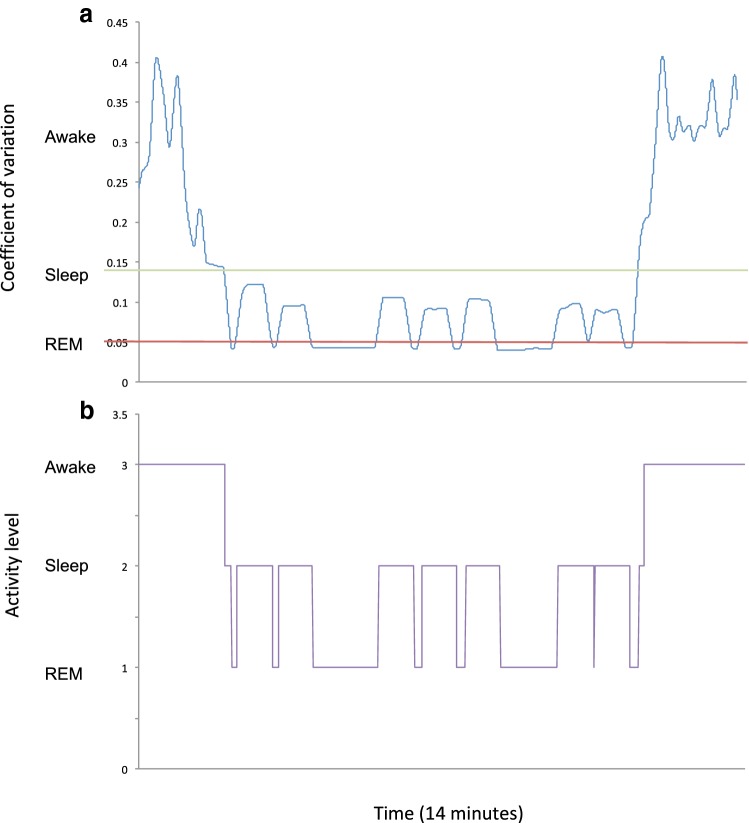
Graphical representation of the various stages of sleep quantified using the WSS. **a** Represents the combined coefficient of variation from all three sensors; **b** represents the separation of (**a**) into the three phases: 1 (REM), 2 (sleep) and 3 (awake)

Activity classification across five participants estimated 28.5% of the time spent awake (28.6% simulated), 42.6% of the time spent asleep (42.9% simulated) and 26.6% of the time spent in REM (28.6% simulated). The activity measured for the simulated sleep of one participant are shown in Fig. 4, represented as the combined coefficient of variation calculated from each of the 3 accelerometer axes of each sensor.

## Discussion

**Fig. 5:**
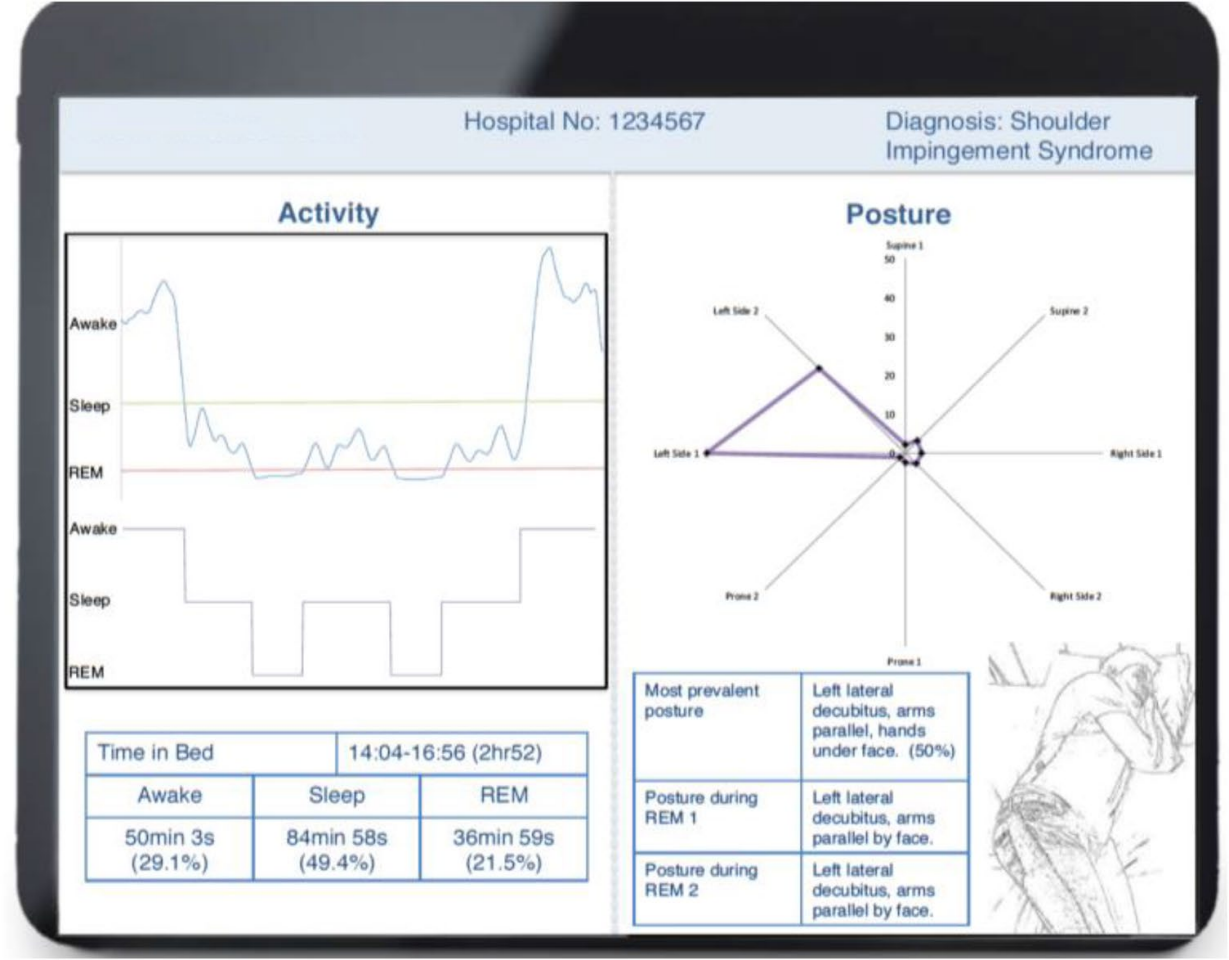
Proof of concept output from the pervasive sleep sensor platform presented as part of a conceptual application interface. Reported data include demographics, activity levels with corresponding time intervals and relative posture for utilisation by clinicians and patients

This study demonstrates the high accuracy levels achievable for monitoring physical activity and postures during sleep with a wearable sensor platform. The WSS was found to have an overall accuracy of 99.5% in the detection of four main postures, which was mostly maintained when detecting eight postures producing an accuracy of 92.5%. Accuracy was acceptable across all subjects with the least accurate being 84.3%. The platform could predict simulated sleep phases (awake, sleep, REM) using arm and trunk activity measurements. The ability of the system to detect both posture and activity was exhibited in a proof of concept dataset, along with a conceptual layout for a tablet application to be used by both doctors and patients (Fig. 5).

The 4-posture classification accuracy (99.5%) compares well to other papers in the field. Hsia et al. [23] used a Bayesian Classification with pressure sensors finding an overall accuracy of 81.4%. The use of a wireless identification and sensing platform by Hoque et al. [36] gained a 94.4% accuracy in detection of the four postures, whilst Ni et al. [27] received similar results to this study in their use of UWB tags combined with a pressure sensor matrix, at approximately 99% accuracy. For eight postures, the WSS demonstrated an accuracy of 92.5%. Two studies using embedded pressure sensors, one considering five postures, and the other nine, gained accuracies of 97.7 and 94%, respectively [24, 25]. Although both these papers gained a higher classification accuracy compared to our 92.5%, pressure systems were embedded within mattresses, with the system described remaining the most accurate and advanced wearable platform.

Distinction between two right-sided postures (1 and 2) yielded the greatest identification error during our study. These postures are very similar, with the only difference being 90° rotation at the shoulder in one arm. Therefore, it is possible that the error in these postures is due to poor user compliance with the participant not recreating the posture performed during calibration. These findings are replicated on the left side. The possibility of poor participant compliance is further highlighted by the fact that in 2 out of 10 participants the sensors were 100% accurate, whilst other participants had accuracy levels as low as 84.3%. That said, in a true clinical model similar issues are likely to occur, but whether minor differences in position will affect the utility of the information in currently unclear.

The WSS could measure the duration of each sleep phase with high accuracy, only the REM phase having a greater than 1% deviation from the predicted, leading to an overall accuracy of 97.3%. This validates the ability of WSS to assess activity levels of a person whilst they are sleeping, facilitating the assessment of sleep quality in a natural environment. This compares well to the actigraph sensor, with the added benefit of simultaneous posture detection. Chang et al. [37] are the only other group who have been able to provide a platform that is able to detect both sleeping posture and activity levels. A tri-axial accelerometer was used on the chest for posture detection, combined with ECG recording for sleep stage monitoring [37]. The method described in this paper provides additional information regarding the position of the upper limbs which is of particular interest in musculoskeletal pathology.

The envisaged clinical impact of the WSS is primarily diagnostic, but with small adjuncts could become therapeutic. Widening access of sleep monitoring beyond specialist facilities would allow patients suffering from upper limb symptoms to consider if sleep position may be a contributing factor. It also facilitates research into the sleep behaviour of post-operative patients, which may give insight into why some experience delay in recovery. The addition of an alert function based on pre-set criteria such as sleeping in one position for too long, might allow for therapeutic utility, e.g. following shoulder arthroplasty. In the broader healthcare setting use of such systems might help prevent pressure sores by alerting carers when patients have been in one position for a certain length of time.

The interpretation and application of the results should be done so in the context of the study limitations. Despite the promising accuracy demonstrated by the WSS, our data were only collected from a cohort of healthy patients measured over 14 min of simulated sleep. Future comparative studies seeking to replicate such findings in a cohort of patients with shoulder pathology over a natural sleep cycle would prove useful in determining if similar results can be obtained in those with shoulder pain and concurrent sleep disturbance. Of note, the sleep postures chosen were designed to capture potential movements replicated in a clinical cohort. Future comparative studies will yield valuable data regarding actual preferred sleep positions in patients with shoulder pathology. As a validation study, the current work proves useful in providing preliminary data to inform the design of future comparative studies.

The main technical limitations of the WSS include battery life and sensor size. Unfortunately, the current WSS battery only lasts for 30 min, making overnight use currently unfeasible. To mitigate this, protocols were tailored accordingly, allowing the simulation of sequences to represent part of a night’s sleep. As prototypes, the sensors are cumbersome (20 × 30 × 17 mm) leading to potential discomfort. Both limitations could be overcome with formal sensor design and packaging, and optimising sensor settings with regard to frequency of data capture and transmission. The study participant demographic was not in keeping with that of the clinical cohort; however, this is unlikely to affect the potential utility.

## Conclusions

This work demonstrates the accuracy of a wireless sensor platform to detect sleeping posture and quality. This has potential for use in patients with musculoskeletal pathology, as well as other healthcare applications such as pressure sore prevention. Ultimately, it is hoped that such sensor platforms could provide a low cost, mobile sleep laboratory, which could facilitate a greater number of sleep studies offering insight into disease processes, and help tailor the holistic management of patients with musculoskeletal disease.

## Additional file

**Additional file 1.** Posture classification algorithm. **(A)** The main part of the algorithm that runs in MATLAB® used to determine posture classification. **(B)** A brief mechanistic overview of the algorithm.

## Abbreviations

WSS: wearable sleep system
REM: rapid eye movements
PSG: polysomnography
UWB: ultra-wide band
HRV: heart rate variability
ECG: echocardiogram
KW-ANOVA: Kruskal–Wallis one-way non-parametric analysis of variance
