# Supplementary figures and images for: A lightweight sensing platform for monitoring sleep quality and posture: a simulated validation study
Source: Eur J Med Res. 2018 May 30;23:28. doi: 10.1186/s40001-018-0326-9 (PMC5975552; doi:10.1186/s40001-018-0326-9)

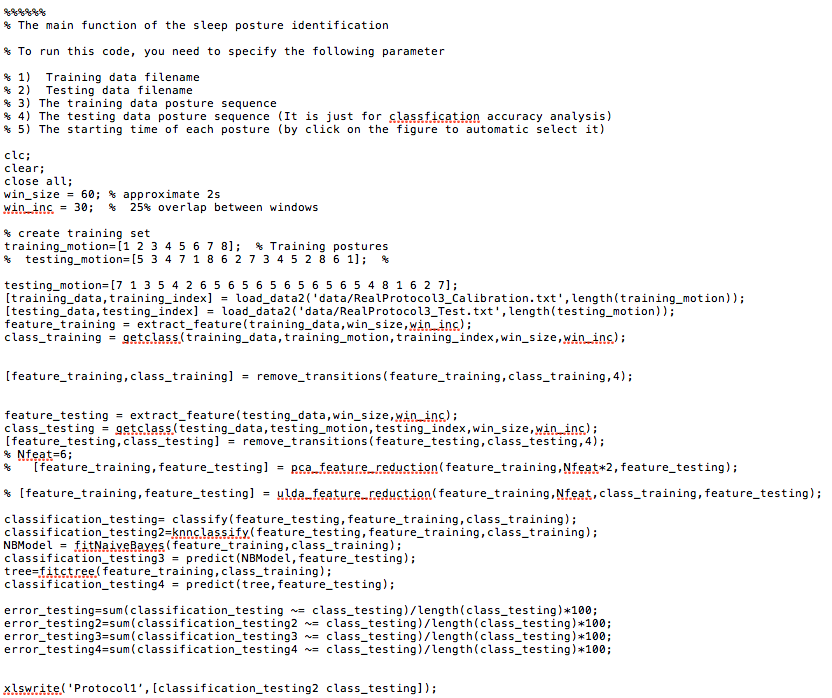


**A**

**B**

Supplement: Supplementary file 1 — Additional file 1. Posture classification algorithm. (A) The main part of the algorithm that runs in MATLAB® used to determine posture classification. (B) A brief mechanistic overview of the algorithm. [file 40001_2018_326_MOESM1_ESM.docx]
